# Comparative assessment of determinants of health-related quality of life in hypertensive patients and normal population in south-west Nigeria

**DOI:** 10.5414/CP202257

**Published:** 2015-01-23

**Authors:** Aduragbenro D.A. Adedapo, Onyinye O. Akunne, Babatunde O. Adedokun

**Affiliations:** 1 Department of Pharmacology and Therapeutics, and; 2 Department of Epidemiology and Medical Statistics, University of Ibadan, Ibadan, Oyo State, Nigeria

**Keywords:** quality of life, hypertension, Africa

## Abstract

Objective: Health-related quality of life (HRQOL) in hypertensive patients may be influenced by the presence and the knowledge of disease, beliefs associated with the disease, blood pressure (BP) control, and drug utilization. The impact of hypertension on HRQOL in hypertensive patients compared to the normal population has not been assessed in Nigeria, the most populous country in sub-Saharan Africa. This study compares HRQOL in hypertensive patients and the normal population; the effect of BP control and medication on HRQOL of hypertensive patients is also assessed. Materials and methods: A prospective cross-sectional study of 713 individuals, 606 were hypertensive patients attending the University College Hospital in Oyo State, Nigeria, while 107 were normal persons residing in Ibadan. Data on sociodemographic status, clinical variables, and drug utilization were collected. World health organization-quality of life short version (WHO-QOL-BREF) questionnaires were used to assess HRQOL of participants. Results: Hypertensive patients had poorer HRQOL compared with normal individuals in the physical health (p < 0.05), psychological (p < 0.01), and total quality of life domains. Blood pressure control had no effect on HRQOL in domain (p > 0.05). Drug use significantly worsened HRQOL of hypertensive patients in the psychological (p ˂ 0.01), social relationship (p < 0.01), and the total quality of life domains (p < 0.01). Multiple regression analysis showed that while income per month was positively predictive of physical, psychological, and total quality of life domains (r^2^ = 1.988, p=0.001; r^2 ^= 3.710, p < 0.001; r^2^ = 2.748, p < 0.001), symptom count was negatively predictive of the same (r^2 ^= –0.746, p = 0.005; r^2 ^= 1.869, p < 0.001; and r^2 ^= –1.094; p < 0.001), respectively. Reduced symptoms and higher income improved quality of life in hypertensive patients. Conclusion: The presence of hypertension and antihypertensive medication reduced HRQOL of hypertensive patients, although BP control surprisingly did not impact HRQOL. However, lower symptom count and higher income improved quality of life.

## Introduction 

Health-related quality of life (HRQOL) of individuals may be affected in most disease conditions, particularly chronic, life-long diseases such as hypertension. Knowledge of the disease as well as medication contributes significantly to the deterioration of HRQOL [1]. Hypertension, the most common noncommunicable disease affecting ~ a billion of the world’s population, is the most important risk factor in the development of cardiovascular and renal diseases [2], with many of the individuals being unaware of their status [3]. Individuals from African descent are mostly affected by this disease because of genetic predispositions [4]. The goal of drug therapy should not only be to improve clinical outcomes of diseases but also improve the quality of life of patients. 

Various instruments are available to assess HRQOL of individuals across cultures. The WHOQOL-BREF questionnaire, one of such instruments, has been vigorously tested cross-culturally to ensure its validity and reliability [5]. HRQOL studies carried out in the hypertensive population suggest low quality of life when compared to normotensive individuals [6, 7, 8]. However, there are inconsistent reports of HRQOL among hypertensive patients with controlled blood pressure (BP) and those with uncontrolled BP; while some reported no significant difference [9, 10], others [11, 12, 13] showed better quality of life in patients with controlled BP. The presence of complications/comorbidities such as diabetes and cardiovascular complications further reduces HRQOL of hypertensive patients [14, 15, 16, 17]. Although Nigeria, as with other sub-Saharan Africa countries, is encumbered with diverse communicable diseases, such as malaria, tuberculosis, and HIV, the current rise in the hypertensive population could lead to a decline in HRQOL of the general populace, negatively impacting socio-economic growth of the country. 

In Africa, limited studies [12] have been carried out to assess the HRQOL in hypertensive patients, and none have been done to assess any discrepancies between hypertensive patients and normal individuals, although the hypertensive population in these developing countries, particularly in urban regions, is comparable to that in developed countries. 

This study compares the HRQOL in hypertensive patients with a reference population, determining the extent to which HRQOL is affected by the presence of the disease. The effect of BP control, antihypertensive drugs prescribed, and presence of complications/comorbidities on HRQOL of hypertensive patients were also studied. 

## Materials and methods 

This was a prospective analytical crosssectional study, carried out at the University College Hospital Ibadan, Oyo state, the foremost tertiary institutional in Nigeria, located in the south west of the country. The health-related quality of life (HRQOL) between individuals diagnosed with hypertension and normotensive individuals were compared. Normotensive individuals were from the general population resident in Ibadan. This study was carried out daily for a period of 6 months. Consecutive hypertensive patients at any stage of hypertension, with or without complication/comorbidity, attending the out-patient clinics of the University College Hospital, Ibadan, and healthy individuals residing in Ibadan were recruited for the study after obtaining informed consent. Ethical approval was given by the University of Ibadan/University College Hospital (UI/CH) Joint Ethical Committee. 

All participants were between the ages of 30 and 85 years. Patients willing to participate present on each clinic day, and normotensive individuals residing in Ibadan were included. Only hypertensive patients who were adherent, i.e., kept appointments and followed drug prescription were studied. 

Sociodemographic data (age, gender, religion, marital status, type of family, educational training, type of job, and monthly income) and data on clinical variables of hypertensive patients were also obtained following a preformed template. Clinical variables studied included number of diagnoses, body mass index [18], history of alcohol intake and smoking, prevalence of symptoms, number of antihypertensive drug prescribed per day, BP control as stipulated by the World Health Organization/International Society of Hypertension (WHO/ISH) guidelines [19] on hypertension management. 

A modified WHO-BREF quality of life questionnaire [5] was used to measure the HRQOL of participants. The questionnaire is divided into four main domains designated to assess the physical, psychological, social relationship, and environmental status of an individual. It is scored on a final scale of 0 – 100, with 0 being the worst possible health status, and 100 the best. Questionnaires were distributed by trained research assistants; verbal translation into the local dialect (Yoruba) was done by the assistants for participants with poor command of English. 

Only questionnaires that were ≥ 80% complete were analyzed. Descriptive statistics were used to analyze sociodemographic details of all participants and clinical data of hypertensive patients. The mean scores of participants in all domains of the WHO-BREF questionnaires were calculated with standard deviations, Mann-Whitney U-test was used to ascertain the significance between the mean values of HRQOL of hypertensive and non-hypertensive participants. The effect of BP control and antihypertensive drug classes on HRQOL of hypertensive individuals was studied using Independent Samples t-test. Statistical significance was defined as p < 0.05. Multiple linear regression analysis was used to test association between HRQOL domains and sociodemographic/clinical variables. SPSS (version 17) (Chicago, IL, USA) was used for data analysis. 

## Results 

A total of 780 questionnaires were distributed to individuals who met the inclusion criteria; 752 were recovered giving a recovery rate of 96.4%. Out of the 752 questionnaires, 713 were included for final analysis as they were 80% complete. Hypertensive patients made up 85.1% (606) of the study participants, while individuals not diagnosed with the disease made up 14.9% (107). The mean age of participants was 57.1 ± 12.9 years, with most individuals between the ages of 60 and 69 years. The female to male ratio was 1 : 0.85, male 45.9% (327), and female 54.1% (386). Table 1 shows the demographic distribution of hypertensive and normotensive participants. Hypertensive patients had a greater female to male ratio, were significantly older and less educated than normotensives (p < 0.01). Most participants (82.8%) received N100,000 (US$ 610) or less per month as income. 

Multiple diagnoses were seen in ~ 31.2% of patients. The most common comorbidities/complications observed was hypertensive heart disease and diabetes. Calcium channel blockers (CCBs) + angiotensin converting enzyme inhibitor (ACEI) + diuretics (D) was the most commonly prescribed drug combination. Single-drug therapy was observed in 11.6% (70) of the hypertensive participants, while 25.9% (157) were not on drug therapy but lifestyle modification. The rest of the hypertensive participants were on multiple-drug therapy, with some receiving as many as four different classes of antihypertensive drugs. Most hypertensive individuals (61.9%, n = 375) had poorly controlled BP, only 38.1% (231) had optimal BP control. Most patients complained of breathlessness (31.8%), while other symptoms, such as dizziness, hearing problems, headache, fatigue, cough, fever, and altered taste, were prevalent among hypertensive participants (Table 2). Most of the patients claimed never to have smoked (98.7%) or consumed alcohol (93.2%). 

The mean total quality of life score of all participants was 64.16 ± 9.15. Participants “without hypertension” had a significantly higher HRQOL in the physical health (Z = –2.20, p = 0.028 Sig < 0.05 2 tailed), psychological (Z = –6.49, p = 0.00 Sig < 0.01 2 tailed), and total HRQOL domains T_QOL (Z = –3.34, p = 0.000 Sig < 0.01 2 tailed) than those diagnosed with hypertension (Table 3). There was no significant difference in HRQOL, in any domain between patients with optimal BP control and those with uncontrolled high BP (Table 3). In the psychological, social relationships, and total quality of life (T_QOL) score domains, hypertensive patients on antihypertensive drugs had significantly lower HRQOL (p < 0.01) than those not on drug therapy (Table 4). Sex, marital status, type of family, symptom count, and educational status had influence on the psychological domain score of participants (Table 5), while total quality of life was affected by type of family, symptom count, and educational status (Table 6). 

Regression analysis showed that while income per month was positively predictive of physical, psychological, and total quality of life domains (r^2^ = 1.988, p = 0.001; r^2 ^= 3.710, p < 0.001; r^2^ = 2.748, p < 0.001), symptom count was negatively predictive of the same (r^2 ^= –0.746, p = 0.005; r^2 ^= 1.869, p < 0.001; and r^2 ^= –1.094; p < 0.001), respectively. Thus, the predictive analysis of variables against domains showed that increasing symptom count was a significant negative predictor of mean quality of life score in all domains (p < 0.01), while increasing income of individuals per month was a significant positive predictor of quality of life scores in all domains (p < 0.01) except the social relationship domain (Table 7). The environment domain recorded the highest positive predictive value with income per month, r^2^ = 4.500, p < 0.001. 

## Discussion 

Healthrelated quality of life has been found to be poorer among individuals with hypertension compared to individuals without the ailment. This is largely thought to be caused by labelling effect of the disease since hypertension is mostly asymptomatic, and adverse effect of drugs used in treatment could contribute to a poorer quality of life among patients. In this study, in the physical (p < 0.05), psychological (p < 0.01), and total quality of life (p < 0.01) domains, individuals with hypertension had a significantly poorer HRQOL than those without hypertension. This finding corraborates results from a systematic study [6] carried out by Trevisol and colleagues, in which meta-analysis of 20 studies revealed a lower quality of life in the physical (–2.43; 95% confidence interval (CI) from –4.77 to –0.0) and mental (–1.68; 95% CI from –2.14 to –1.23) domains. Quality of life in hypertensive individuals was also shown to be slightly worse than normotensives. Blood pressure control was poor among patients but had no significant effect on quality of life in patients in any of the domains; comparable quality of life between patients with controlled BP, and those without controlled BP is a strong indication that individual beliefs, and attitudes, and the absence of the disease contributes to the measure of quality of life, affecting it positively or negatively. This finding differs from a previous study, which showed better quality of life with reduction of BP [11, 12, 13]. 

Most patients had hypertension alone (68.8%), although some had 3 or more comorbidities/complications: hypertensive heart disease, and diabetes mellitus were more common. The presence of comorbidity/complication had no significant effect on quality of life of hypertensive patients. This differs from other studies, in which the presence of diabetes mellitus and heart diseases with hypertension significantly reduced the quality of life of patients [14, 15, 16, 17]; our findings could be the result of minimal severity of the complications/comorbidities in patients. Antihypertensive drugs affect quality of life of patients [7]: Patients on antihypertensive medication had significantly lower quality of life in the psychological (p < 0.01), social relationship (p < 0.05), and total quality of life (p < 0.05) domains than patients not on drug therapy. A study by Erickson and colleagues [8] showed that hypertensive patients on medication had worse quality of life profiles compared to those not on drugs. The effect of side effects of drugs on quality of life among patients with chronic diseases is an important factor to consider since it impacts management of the disease; patients experiencing poorer quality of life as a result of drugs prescribed may feel reluctant to adhere to prescription [20], leading to exacerbation of the disease. 

Higher quality of life score was observed among single male hypertensive patients, with no symptoms; this suggests that individuals with no symptoms had a better total quality of life. Multiple regression results showed that income per month and symptom count significantly relates to quality of life score in all domains except the social relationship domain. Increasing income causes a concomitant increase in the physical (p < 0.01), psychological (p < 0.01), environment (p < 0.01), and total quality of life (p < 0.01) domains. Most (82.8%) of the study participants did not earn more than N100,000, (US$ 610), while 33.9% earned less than N20,000 (US$ 122 at the current exchange rate of N164 to US$1). This could be a contributory factor to the slightly above average quality of life experienced by individuals. Number of symptoms reported by patients had a significant negative relationship to quality of life scores in all domains (p < 0.01). Higher symptom count potentially decreases quality of life of patients, this is similar to previous findings in which increasing symptom count in patients was strongly associated with poorer quality of life [8, 12]. 

The effect of diagnosis on hypertensive patients in this region is consistent with findings in other regions, but the effect of BP lowering on health-related quality of life is inconsistent with other studies. The negative impact of drug use and symptoms which may be the result of disease or adverse effect of drugs, was confirmed in this study. Drugs offered to patients should have a low side effect profile with maximum benefits. Antihypertensive medications with a high side effect profile could be detrimental to the quality of life of patients. This study contributes to quality of life in hypertension, as research on hypertension, and hypertensive medication on hypertensive patients is limited in the sub-Saharan Africa region. 

## Conclusion 

High BP does not influence health-related quality of life of patients, rather, quality of life is influenced by the knowledge of the disease. Presence of symptoms and low income worsen quality of life, antihypertensive drugs lower quality of life significantly. Reorientation of patients’ belief and attitude toward hypertension is needed. Individualized care of patients, with drugs having low adverse effect profile, is recommended. 

## Acknowledgment 

We acknowledge Professor Babatunde Salako of the Nephrology unit, Dr. Olulola O Oladapo of the Cardiology Clinic, and Dr. Achiaka Irabor of the Family Medicine Department of the University College Hospital for their assistance and collaboration. We also want to thank Mrs. Fisayo Adeyemo for her assistance with data entry. The staff of the medical records department of the University College Hospital Ibadan, and all who contributed to this research, are appreciated. 

## Conflict of interest 

The authors declare no conflict of interest. 

**Table 1.** Sociodemographic variables of hypertensive and nonhypertensive subjects in health-related quality of life study in a sub-Saharan African region.

Characteristics	Total (%)	Hypertensives (%)	Normotensives (%)	*p-value
	n = 713	n = 606	n = 107	
Age (years)				
30 – 39	0.8	3.4	67.6	0.00
40 – 49	17.3	16.8	20.3	
50 – 59	23.8	26.5	4.1	
60 – 69	28.6	31.7	5.4	
70 – 79	16.4	18.2	2.7	
≥ 80	3.1	3.4	0	
Gender				
Male	45.9	44.2	55.2	0.27
Female	54.1	55.8	44.8	
Religion				
Christianity	69.4	66.6	85.6	
Islam	30.3	33.2	13.5	
Others	0.3	0.2	1.0	
Marital status				
Single	5.6	1.7	28.3	0.00
Married	78.0	79.3	70.8	
Divorced	2.9	3.5	0	
Widowed	13.5	15.5	0.9	
Type of family				
Monogamy	76.9	74.8	90.4	0.00
Polygamy	23.1	25.2	9.6	
Highest educational level				
None	11.6	13.5	1.9	0.00
Primary	43.3	47.3	21.9	
Secondary	12.6	13.8	5.7	
Tertiary	32.4	25.4	70.5	
Job category				
White-collar	28.9	22.8	65.6	0.00
Self-employed	37.0	39.9	19.4	
Blue-collar	7.7	8.1	5.4	
Unemployed/retired	26.4	29.2	9.7	
Income per month (n) Naira.				
Less than 20,000	33.9	36.0	23.5	0.01
20,000 – 100,000	48.9	48.1	52.9	
101,000 – 250,000	12.2	11.4	16.7	
More than 250,000	4.9	4.5	6.9	

*χ^2^-test (level of significance p < 0.05).

**Table 2.** Clinical variables of hypertensive patients in health-related quality of life study in a sub-Saharan African region.

Clinical variable	Frequency	Percentage (%)
No. of diagnoses		
1	417	68.8
2	163	26.9
≥ 3	26	4.3
Blood pressure control		
Not controlled	375	61.9
Controlled	231	38.1
BMI*		
< 18.50	8	1.5
18.50 – 24.99	213	37.7
25.00 – 29.99	170	30.0
≥ 30	174	30.8
Alcohol intake		
Never	565	93.2
Quite	32	5.3
Often	9	1.5
Smoking		
Never	598	98.7
Quite	2	0.3
Often	6	1.0
Salt		
Little	361	59.6
Moderate	206	34.0
Very much	39	6.4
Prevalence of symptoms		
Breathlessness	193	31.8
Dizziness	151	24.9
Hearing problems	38	6.3
Headache	142	23.4
Fatigue	68	11.2
Cough	76	12.5
Fever	382	63.0
Altered taste	45	7.5
Number of antihypertensive drug per day		
0	157	25.9
1	70	11.6
2	201	33.2
3	150	24.8
4	28	4.6
Complication/co-morbidity		
Hypertensive heart disease	69	11.4
Diabetes mellitus	69	11.4
Renal diseases	18	3.0
Dyslipidemia	8	1.3
Others (arthritis)	49	6

*Missing value = 41.

**Table 3.** Health-related quality of life (HRQOL) scores of hypertensive and nonhypertensive individuals in relation to blood pressure control.

Domain	Total	Hypertensive	Non-hypertensive	Controlled	Uncontrolled
Physical health	59.37 ± 10.94	58.98 ± 10.67	61.63 ± 12.22^+^	59.43 ± 10.23	59.04 ± 10.56
Psychological	68.63 ± 12.72	67.32 ± 12.59	76.02 ± 10.85^++^	67.51 ± 12.02	66.50 ± 12.26
Social relationships	68.71 ± 14.48	68.33 ± 13.90	70.87 ± 17.35	67.48 ± 14.20	68.11 ± 12.91
Environment	59.79 ± 12.59	59.59 ± 12.25	60.93 ± 14.39	58.98 ± 13.03	59.08 ± 11.29
Total quality of life	64.16 ± 9.15	63.59 ± 8.91	67.38 ± 9.83^++^	63.60 ± 8.65	63.24 ± 8.47

(T_QOL) score, Mann-Whitney U-test, ^+^p < 0.05, ^++^p < 0.01.

**Table 4.** Health-related quality of life (HRQOL) score of patients on drug therapy vs. patients not on drug therapy.

	Domain patients on drug therapy	Patients not on drug therapy	p-value
Physical health	59.26 ± 10.37	59.56 ± 11.88	0.73
Psychological	67.14 ± 12.12	71.16 ± 13.33	0.00^##^
Social relationships	67.67 ± 13.58	70.50 ± 15.79	0.01^#^
Environment	59.21 ± 12.20	60.78 ± 13.19	0.11
Total quality of life	63.49 ± 8.53	65.32 ± 10.03	0.01^#^

(T_QOL) score. Independent-Samples t-test, ^#^p < 0.05, ^##^p < 0.01.

**Table 5.** Influence of variables on psychological domains.

Variables sub-group	Mean ± SD	Test statistics	p-value
Sex			
Female	67.40 ± 12.43	–1.300	0.006
Male	70.02 ± 12.94		
Marital status			
Single	75.69 ± 13.41	0.471	0.001
Married	68.73 ± 12.66		
Type of family			
Monogamy	69.11 ± 12.37	2.532	0.001
Polygamy	65.98 ± 13.01		
Symptom count			
0	73.44 ± 11.11	3.750	0.000
≥ 1	67.976 ± 12.80		

**Table 6.** Influence of variables on total QOL score domains.

Variables sub-group	Mean ± SD	Test statistics	p-value
Type of family			
Monogamy	64.53 ± 9.10	2.702	0.008
Polygamy	62.28 ± 8.52		
Symptom count			
0	67.52 ± 9.57	3.580	0.000
≥1	63.70 ± 9.00		

**Table 7.** Multiple regressions relating quality of life scores in each domain to predictor variables.

Domain variables	Coefficient	Beta	p-value
Physical health			
Age	–0.055	–0.061	0.268
Religion	0.586	0.028	0.557
Marital status	0.918	0.066	0.176
Type of family	–0.327	–0.014	0.787
Job category	–0.226	–0.025	0.631
Income per month	1.988	0.158	0 .001**
Symptom count	–0.746	–0.128	0.005**
Psychological			
Age	–0.027	–0.026	0.620
Religion	–0.939	–0.040	0.386
Marital status	1.144	0.071	0.123
Type of family	–1.333	–0.048	0.314
Job category	–0.539	–0.053	0.290
Income per month	3.710	0.258	0.000**
Symptom count	–1.869	–0.278	0.000**
Social relationships			
Age	0.026	0.022	0.690
Religion	–0.558	–0.021	0.666
Marital status	–2.256	–0.123	0.011**
Type of family	–2.411	–0.077	0.126
Job category	–1.002	–0.085	0.101
Income per month	1.362	0.083	0.081
Symptom count	–0.943	–0.123	0.006**
Environment			
Age	0.134	0.121	0.021*
Religion	–3.439	–0.138	0.003**
Marital status	0.892	0.053	0.260
Type of family	–0.418	–0.014	0.767
Job category	0.514	0.047	0.346
Income per month	4.500	0.298	0.000**
Symptom count	–0.779	–0.110	0.011**
Total HRQOL score			
Age	0.024	0.031	0.555
Religion	–1.154	–0.067	0.154
Marital status	0.219	0.019	0.694
Type of family	–0.908	–0.045	0.360
Job category	–0.231	–0.031	0.542
Income per month	2.748	0.264	0.000**
Symptom count	–1.094	–0.224	0.000**

*p < 0.05, **p < 0.01.
